# Serum antibody profiles in individuals with latent *Mycobacterium tuberculosis* infection

**DOI:** 10.1111/1348-0421.12674

**Published:** 2019-04-09

**Authors:** Ryoji Maekura, Seigo Kitada, Mayuko Osada‐Oka, Yoshitaka Tateishi, Yuriko Ozeki, Takeya Fujicawa, Mari Miki, Ogawa Jyunnko, Masahide Mori, Sohkichi Matsumoto

**Affiliations:** ^1^ Department of Respiratory Medicine National Hospital Organization Toneyama National Hospital Toyonaka Osaka Japan; ^2^ Graduate School of Health Care Sciences, Jikei Institute Osaka Japan; ^3^ Food Hygiene and Environmental Health, Division of Applied Life Science, Graduate School of Life and Environmental Sciences, Kyoto Prefectural University Kyoto Japan; ^4^ Department of Bacteriology Niigata University Graduate School of Medicine Niigata Japan

**Keywords:** Biomarker, latent tuberculosis infection, mycobacterial DNA‐binding protein 1, serum antibody

## Abstract

One‐third of the world's humans has latent tuberculosis infection (LTBI), representing a large pool of potentially active TB. Recent LTBI carries a higher risk of disease progression than remote LTBI. Recent studies suggest important roles of antibodies in TB pathology, prompting us to investigate serum antibody profiles in a cohort with LTBI. In this single‐center prospective observational study, we analyzed IgG‐antibody concentrations against five major *Mycobacterium tuberculosis* (*Mtb*) antigens (including 6 kDa early secretory antigenic target (ESAT6), CFP10, and antigen 85A, which are expressed mainly in the growth phase; and mycobacterial DNA‐binding protein 1 (MDP1) and alpha‐crystallin like protein (Acr), which are expressed in the dormant phases) in individuals with recent (*n*=13) or remote (*n*=12) LTBI, no *Mtb* infection (*n*=19), or active TB (*n*=15). Antibody titers against ESAT6 and MDP1 were significantly higher in individuals with recent LTBI than in those with no *Mtb* infection or remote LTBI. All pairwise antibody titers against these five major antigens were significantly correlated throughout the stages of *Mtb* infection. Five individuals with recent LTBI had significantly higher antibody titers against ESAT6 (*P* = 0.03), Ag85A (*P* = 0.048), Acr (*P* = 0.057), and MDP1 (*P* = 0.0001) than in individuals with remote LTBI; they were also outside the normal range (+2 SDs). One of these individuals was diagnosed with active pulmonary TB at 18‐month follow‐up examination. These findings indicated that concentrations of antibodies against both multiplying and dormant *Mtb* are higher in recent LTBI and that individuals with markedly higher antibody titers may be appropriate candidates for prophylactic therapy.

**Abbreviations**
Acr: alpha‐crystallin like protein
CFP: culture filtrate protein
Dos S: sensor histidine kinase of DosR
EIA: enzyme‐linked immunosorbent assay
ESAT: early secretory antigenic target
IGRA: interferon gamma release assay
LAM: lipoarabinomannan
LTBI: latent tuberculosis infection
MAC: *Mycobacterium avium* complex
MDP1: mycobacterial DNA binding protein 1
Mtb: *Mycobacterium tuberculosis*
TB: tuberculosis
TBGL: tuberculous glycolipid

## 1 INTRODUCTION

In 2016, 10.4 million people newly developed tuberculosis (TB), in the vast majority of cases arising from LTBI.^1^ It is estimated that one‐third of all humans have asymptomatic *Mtb*, and are therefore at risk of disease progression. Individuals with past or old TB who have abnormal chest radiographs are at higher risk of disease progression.^2^ Individuals with LTBI are also asymptomatic, but have no abnormalities on chest radiographs. However, LTBI also carries the risk of TB progression depending on the time interval since infection. The American Thoracic Society classifies *Mtb*‐infected individuals as symptomatic or asymptomatic.^2^ They divide the asymptomatic group into the following subgroups: (i) past TB (inactive TB; class 4); (ii) LTBI (class 1), which is further divided into recent LTBI (<2 years since infection) or remote LTBI (>2 years since infection); and (iii) preclinical TB (including asymptomatic individuals who subsequently develop active TB). Identification of people at risk of TB progression is important regarding administration of appropriate preventative medication. However, it is difficult to differentiate recent LTBI or preclinical TB from remote LTBI because of the wide spectrum of poorly characterized clinical features of *Mtb* infection.^3^ Recently validated diagnostic methods, such as IGRAs, do not permit assessments of the risk of TB progression.^4^ Thus, distinguishing between stages of *Mtb* infection remains an important diagnostic challenge.

Recent studies suggest that antibodies play important roles in TB pathology and diagnosis.^5^, ^6^ We have previously reported that antibody titers against the following six of the 23 types of *Mtb* antigens, antigen 85A (Ag85A), 6 kDa ESAT6, 10 kDa CFP10, MDP1, Acr, and sensor histidine kinase of DosR (Dos S), are high in individuals with past TB.^7^ Antigen 85A is expressed in the growth phase of *Mtb*, whereas ESAT6 and CFP10 are expressed in the growth to stationary phases^8^, ^9^ and MDP1, Acr, and Dos S are upregulated in the stationary to dormant phases.^10^ These data suggest the coexistence of both growing and dormant *Mtb* in individuals with past TB and the utility of antibody responses in determining TB infection status. However, it is, still necessary to evaluate biomarker‐targeted antibody responses in individuals with recent LTBI and preclinical TB, both of whom are at higher risk of TB progression than individuals with remote LTBI.

In this study, we conducted a hospital‐based survey at Toneyama National Hospital, Osaka, Japan in which, since 2000, patients with active TB have been receiving chemotherapy for approximately 2 months in a specialized negative‐pressure ward. Although staff always wear N95 masks in this ward, several staff members were infected with *Mtb* and their disease progressed to active TB in 2000‐2010. In 2010, healthcare workers at Toneyama National Hospital underwent extensive contact screening regarding transmission of TB. Individuals considered to have recent or remote LTBI on the basis of their IGRAs in an annual medical examination were selected from all staff members who had been in contact with patients with active TB. Serum antibody profiles against major *Mtb* proteins, including the six above‐listed antigens, were analyzed in individuals with recent LTBI, remote LTBI, no *Mtb* infection, and active TB to identify biomarkers to determine of TB progression.

## 2 MATERIALS AND METHODS

### 2.1 Subjects

With the aim of preventing nosocomial transmission of TB, IGRAs have been included in periodic medical examinations of healthcare workers since 2010. All staff members with positive reactions to BCG vaccination and who had been in contact with patients with TB underwent annual medical examinations, including chest X‐ray and irregular TB patient contact examinations, from 2011 to 2015. LTBI was diagnosed on the basis of positive IGRAs, with no clinical, bacteriological, or radiographic evidence of active disease. Individuals with LTBI were further categorized as having recent or remote LTBI according to the results of annual and irregular medical examinations. IGRAs to identify recent LTBI have been assessed yearly in new staff members and staff members who were previously IGRA‐negative. The specimens for serodiagnosis were collected from the staff and inpatients with TB after obtaining written informed consent. This study was approved by the Research and Ethical Committees of the National Toneyama Hospital (2009‐0920) and Osaka City University Graduate School of Medicine (1458).

Participants were divided into the following groups:.

1) Recent LTBI group with positive IGRA (<2 years after infection). This group consisted of 13 individuals (aged 43 ± 10 years, male/female ratio 3/10) with positive conversion of IGRA, including 10 identified as a result of annual medical examinations in 2011 and 2012 and three identified in irregular contact examinations of staff members who had previously been IGRA‐negative.
2) Remote LTBI group (>2 years after infection). This group consisted of 12 individuals (aged 50 ± 6 years, male/female ratio 3/9) who had been IGRA‐positive from 2 years before 2012.
3) No *Mtb* infection group. This group consisted of 19 individuals (aged 44 ± 10 years, male/female ratio 5/14) who were IGRA‐negative results and had negative results on testing with three commercially available serodiagnostic tests (Determiner TBGL kit [Kyowa Medex, Tokyo, Japan], MycoDot kit [Mossman Associates, Blackstone, MA], and MAC EIA kit [Tauns Laboratory, Shizuoka, Japan]).
4) Positive serodiagnosis group. This group consisted of 15 individuals (aged 44 ± 8 years, male/female ratio 5/10) who were IGRA‐negative and positive according to Determiner TBGL and/or MycoDot tests. One individual with a positive MAC EIA test result was excluded from this study.
5) Active TB group. This group included 15 inpatients (aged 47 ± 18 years, male/female ratio 8/7) whose were diagnosed as having active TB by microbiologic examination of sputum specimens yielding positive cultures and positive RNA amplification tests specific for *Mtb* (TRC Test, TRCRapid‐160; Tosoh; Tokyo, Japan) in 2011.

### 2.2 Commercially available IGRAs and serological tests

IGRAs was performed using QFT‐Gold according to the manufacturer's instructions (Cellestis, Carnegie, Australia) with CFP10 and ESAT6 of *Mtb* as antigens and a cutoff value for a positive IFN‐γ test of ≥0.35 IU/mL. Anti‐TBGL antibodies were measured by ELISA using a Determiner TBGL Kit with tuberculous glycolipids, including cord factor as an antigen. Anti‐LAM antibodies were detected using a MycoDot kit. Antibody detection was achieved using representative nitrocellulose discs for dot‐blot assays, the color intensities of the test reactions being compared with those of references. Kits for anti‐LAM and anti‐TBGL antibody testing were specific for IgG responses. The manufacturers' instructions for test performance were followed for all commercially available assays used in this study. Results of the TBGL test are expressed as U/mL and of the LAM test using a 5‐point scale. The cutoff points were 2 U/mL for anti‐TBGL antibodies and 1 + for anti‐LAM antibodies, in accordance with the standards set by the manufacturer of each test. GPL serum core IgA antibody titers were measured using a MAC EIA kit according to the manufacturer's instructions. All specimens were assayed without prior knowledge of the clinical status. Materials and reagents have been described in detail in a previous paper.^7^

### 2.3 Recombinant protein preparation

Recombinant *Mtb* antigens, including ESAT6 (Rv3875), CFP10 (Rv3874), MDP1 (Rv2986c), HBHA (Rv0475), Acr (Rv2031c), HrpA (Rv0251c), DosS (Rv3132), Ag85A (Rv3804c), and Ag85B (Rv1886c), were expressed in *Escherichia coli* BL21 (DE3) using a pET21b vector and purified by affinity chromatography, as described previously.^7^

### 2.4 ELISA

Titers of IgG antibodies against recombinant *Mtb* proteins were determined by ELISA as follows. Ninety‐six well microplates (Sumilon Type H; LMS, Tokyo, Japan) were coated with one of the recombinant antigen in bicarbonate buffer, pH 9.6 (0.5 μg/well) overnight at 4°C. The plates were then blocked with PBS containing 0.05% Tween 20 and 5% skim milk for 12 hours at 4°C and washed four times with PBS containing 0.05% Tween 20. Human serum samples diluted 1:100 in PBS containing 0.05% Tween 20 and 0.5% skim milk were incubated for 12 hours at 4°C. After washing, HRP‐conjugated anti‐human IgG antibodies were added at a 1:5000 dilution. After 1 hour of incubation at 37°C, the plates were washed four times, and 100 μL of SureBlue reserve‐TMB Microwell Peroxidase Substrate (KPL, Gaithersburg, MD) added to each well. The reactions were stopped after 3 minutes by the addition of 50 μL of 0.1 M HCl, after which the plates were read at 450 nm using a Multiskan (Thermo Fisher Scientific K.K., Yokohama, Japan). Samples were analyzed in triplicate. The normal range for each antibody titer was defined as less than two SDs above the mean titer of the group with no *Mtb* infection.

### 2.5 Statistical analyses

Statistical analyses were performed using JMP 9 (SAS Institute, Cary, NC). Antibody titers were obtained by delta OD and are reported as means ± SD. Logarithm‐converted antibody titers were used for statistical analyses and correlations between antibody titers were analyzed. The Kruskal–Wallis test was used to determine differences in antibody titers between groups. Differences between pairs of groups were analyzed using the Tukey–Kramer honest significant difference post hoc test. Relationships between antibody titers were assessed using Pearson correlation coefficients and linear regression analyses. Differences were considered significant when *P* < 0.05. The cut‐off values for positivity of each antibody were defined as two SDs above the mean titer of the group with no *Mtb* infection.

## 3 RESULTS

### 3.1 Enrollment of individuals with recent LTBI

Thirteen individuals with recent LTBI diagnosed on the basis of results of annual medical examinations and irregular screenings from 2011 to 2015 were enrolled (Table 1). No individuals with positive IGRA conversion were identified in 2013, 2014, or 2015. All staff, including those in the recent LTBI group, reported no respiratory symptoms and had no radiographic evidence of current disease. Serum specimens obtained in 2011 and 2012 were used to measure IgG antibody titers against *Mtb* antigens.

**Table 1:** Enrollment of healthcare workers with recent LTBI

		IGRA results			Number of HCWs	Number of TB inpatients/year
	Positive	Positive conversion	Negative	Indeterminate		
Annual examinations						
2010	15 (9.2%)		131 (80.4%)	17 (10.4%)	163	489
2011	9 (6.7%)	5	108 (80%)	18 (13.3%)	135	335
2012	13 (8.7%)	5	117 (78%)	20 (13.3)	150	257
2013	6 (2.9%)	0	199 (94.7%)	5 (2.4%)	210	215
2014	2 (1.1%)	0	178 (96.7%)	4 (2.2%)	184	208
2015	0	0	148 (99.3%)	1 (0.7%)	149	160
Irregular screenings						
2011–2012	3	3	15			

Abbreviations: HCWs: Health Care Workers.

### 3.2 Comparisons of antibody titers

Comparisons of IgG antibody titers against *Mtb* antigens in healthcare workers and inpatients with active TB are summarized in Table 2. There are no significant differences in age between the five groups. Antibody titers against the five selected antigens were significantly higher in patients with active TB than in the group of non‐*Mtb*‐infected persons (*P* < 0.0001). The rates of positivity for antibodies against ESAT6, CFP10, Ag85A, Acr, and MDP1 were 60%, 33%, 67%, 80%, and 80%, respectively. Two patients (13%) were negative results for all five tested antigens. The antibody titers against four antigens, the exception being CFP10, were also significantly higher than those in the recent and remote LTBI groups. However, there was no significant difference in antibody titers against ESAT6 between the active TB and recent LTBI groups.

**Table 2:** Comparison of IgG antibody titers against *M. tuberculosis (Mtb)* antigens between healthcare workers and patients with active TB

			Healthcare workers		
	Active TB	No *Mtb* infection	Recent LTBI	Remote LTBI	Serodiagnosis (+)
Number (M:F)	15 (8:7)	19 (5:14)	13 (3:10)	12 (3:9)	15 (5:10)
Age, y	47 ± 18.1 (23–78)	44 ± 9.8 (27–58)	43 ± 10.3 (24–58)	50 ± 6.4 (38–58)	44 ± 7.5 (28–57)
Antigens	Antibody titers (ΔOD)				
ESAT6	0.49 ± 0.56	0.04 ± 0.02#	0.31 ± 0.41	0.07 ± 0.07*	0.04 ± 0.02$
CFP10	0.48 ± 0.49	0.12 ± 0.1#	0.24 ± 0.24	0.24 ± 0.24	0.21 ± 0.16
Acr	0.84 ± 0.51	0.15 ± 0.07#	0.38 ± 0.28*	0.26 ± 0.15#	0.25 ± 0.18$
MDP1	1.52 ± 0.86	0.27 ± 0.12#	0.86 ± 0.43*	0.33 ± 0.15#	0.38 ± 0.13#
Ag85A	1.93 ± 0.87	0.76 ± 0.29#	1.01 ± 0.87*	1.02 ± 0.44$	0.91 ± 0.47&
QFT‐Gold		(‐):15, ( ± ):4, ( + ):0	(‐):0, ( ± ):0, ( + ):13	(‐):0, ( ± ):0, ( + ):12	(‐):13, ( ± ):2, ( + ):0
TBGL (unit/mL)		0.20 ± 0.34	1.62 ± 2.06	1.88 ± 2.59	5.08 ± 6.44
LAM		(‐):19, ( + ):0	(‐):9, ( + ):4	(‐):9, ( + ):3	(‐):5, ( + ):10
GPL‐core (unit/mL)		0.01 ± 0.02	0.05 ± 0.05	0.01 ± 0.01	0.03 ± 0.03

Abbreviations: ΔOD, optical density (average ± SD); M:F, male:female.

P‐values between active TB and other groups:

ESAT6 (#: *P* = 0.0006, *: *P* = 0.0071, $: *P* = 0.0014)

CFT10 (#: *P* = 0.0033).

Acr (#: *P* < 0.0001, *: *P* = 0.0005)

MDP1 (#: *P* < 0.0001, *: *P* = 0.0015)

Ag85A (#: *P* < 0.0001, *: *P* = 0.0017, $: P = 0.0027, &: *P* < 0.0001)

**Figure 1:**
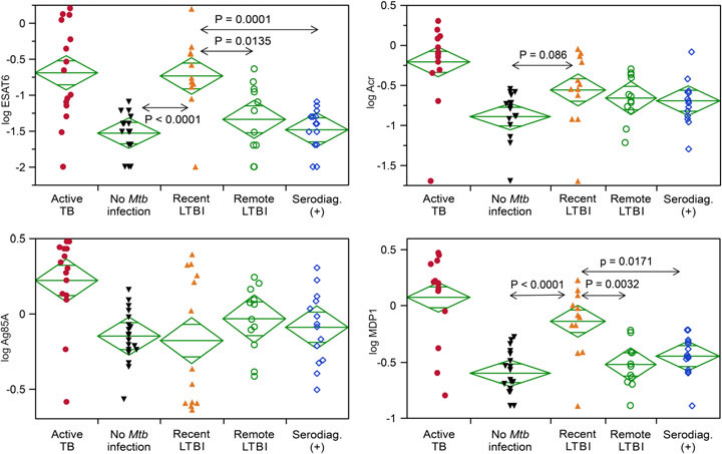
Comparative analysis of IgG antibody titers against four *Mtb* antigens among the five study groups. Individuals with recent LTBI had significantly higher logarithm‐converted antibody titers against ESAT6 (*P* < 0.0001; *P* = 0.0004) and MDP1 (*P* < 0.0001; *P* = 0.0003) than those in the groups with no *Mtb* infection or remote LTBI. ESAT, early secretory antigenic target; LTBI, latent tuberculosis infection; MDP1, mycobacterial DNA‐binding protein 1; *Mtb*, *Mycobacterium tuberculosis* [Color figure can be viewed at wileyonlinelibrary.com]

Individuals with recent LTBI had significantly higher logarithm‐converted antibody titers against ESAT6 than those in the groups with no *Mtb* infection or remote LTBI (*P* < 0.0001; *P* = 0.0004, respectively); the same was true for MDP1 (*P* < 0.0001; *P* = 0.0003, respectively) (Figure 1). In the recent LTBI group, the proportions with antibodies against ESAT6, CFP10, Ag85A, Acr, and MDP1 were 85%, 8%, 38%, 31%, and 77%, respectively. Two patients(15%) had no absorbance for antibodies against any of the five antigens. No increases in antibody titers against other antigens (DosS, HBHA, HrpA, and Ag85B) were detected in the recent LTBI group (data not shown).

### 3.3 Relationships between logarithm‐converted antibody titers against the five selected antigens

**Figure 2:**
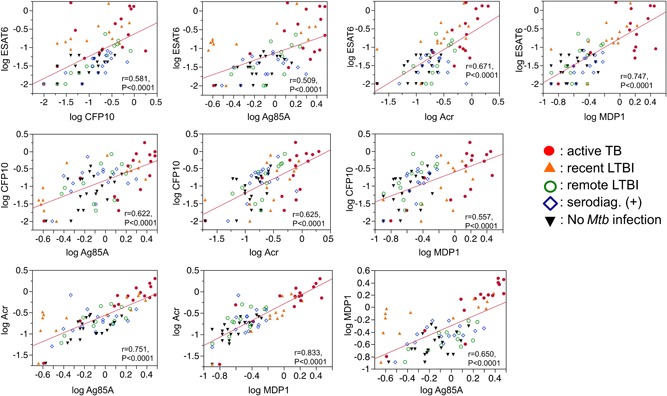
Relationships between logarithm‐converted antibody titers against the five selected antigens in all participants. There were significant correlations between all pairs of antibody titers against ESAT6, CFP10, Ag85A, Acr, and MDP1 (all *P* < 0.0001). Each symbol indicates an individual from one of the five groups [Color figure can be viewed at wileyonlinelibrary.com]

There were significant correlations between all pairs of antibody titers against ESAT6, CFP10, Ag85A, Acr, and MDP1 (all *P* < 0.0001) (Figure 2). Although some antibodies were not detected in some patients, the antibody titers in patients with active TB were the highest (right‐upper area in Figure 2), followed by the antibody titers in individuals with recent LTBI. The antibody titers of individuals in the group with no *Mtb* infection were generally low.

### 3.4 Detection of preclinical TB

**Figure 3:**
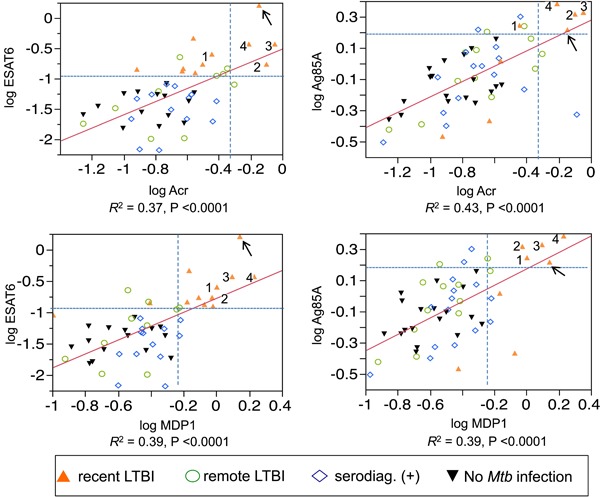
Antibody titers against four antigens are shown for all healthcare workers, except for those whose antibody titers were below the detection level. The cutoff values (dotted lines) for positivity were defined as two SDs above the mean titer in the group without *Mtb* infection. Arrow indicates an individual with preclinical TB. Numbers 1, 2, 3, and 4 indicate cases 1, 2, 3, and 4 in the recent LTBI group, respectively. In these five individuals, the antibody titers against ESAT6 (*P* = 0.03), Ag85A (*P* = 0.048), Acr (*P* = 0.057), and MDP1 (*P* = 0.0001) were significantly higher than those in the remote LTBI group [Color figure can be viewed at wileyonlinelibrary.com]

**Figure 4:**
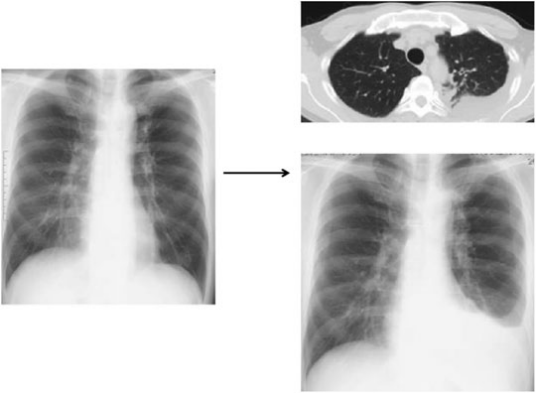
Detection of preclinical TB**.** An individual with recent LTBI was found to have progressed to having active pulmonary TB with pleurisy 19 months after measurement of serum antibody titers. Log‐corrected antibody titers for ESAT‐6, CFP10, MDP1, Acr, and Ag85A were 0.2, −0.57, 0.14, −0.14, and 0.22, respectively

In five individuals with recent LTBI (including the one indicated by an arrow and case numbers 1, 2, 3, and 4 in Figure 3), antibody titers against ESAT6 (*P* = 0.03), Ag85A (*P* = 0.048), Acr (*P* = 0.057), and MDP1 (*P* = 0.0001) were significantly higher than those in the remote LTBI group. These antibody titers were also above their normal ranges. The individual preclinical TB indicated with an arrow in Figure 3 was diagnosed as having active pulmonary TB with pleurisy by chest X‐ray examination 19 months after measurement of serum antibody concentrations (Figure 4).

## 4 DISCUSSION

To identify biomarkers of TB progression in asymptomatic individuals, we conducted a hospital‐based survey and compared biomarker‐targeted antibody responses between individuals with recent LTBI and remote LTBI, the former being at higher risk of TB progression than the latter.^2^ For controls, we also assessed sera of TB patients and staff members with no *Mtb* infection. Negative IGRA results alone are not sufficient to conclude there is no *Mtb* infection, as indicated by some patients with active TB reportedly being negative for IGRA.^11^ Therefore, to avoid false negatives in the present study, the group with no *Mtb* infection was required to be negative according to three commercially available serodiagnostic tests in addition to being negative for IGRA. However, we did not determine the sensitivity and specificity for individual antibodies because there were too few individuals with no *Mtb* infection (negative controls) and hospital staff are always at risk of infection with acid‐fast bacteria. Although TB‐specific antigens expressed in the growth or dormant phases are required for an accurate diagnosis of recent LTBI, ESAT6 and CFT10 are present in *Mycobacterium kansasii* and MDP1 is present in *M. avium* and other mycobacteria. The sensitivity of each anti‐TB antibody is not high enough to make a definite diagnosis of recent LTBI. Therefore, a diagnostic antibody panel using a combination of multiple antigens (TB‐specific antigens, nontuberculous mycobacteria [NTM]‐specific antigens, latent infection‐related antigens, and common antigens) is necessary for the identification of appropriate candidates for prophylactic therapy.^11^

In our study, antibody titers against ESAT6 and MDP1 were significantly higher in recent LTBI than in remote LTBI. Several individuals in the recent LTBI group had high antibody titers against Ag85A or Acr. The antibody titers to DosS, HBHA, HrpA, and Ag85B were under the detection limits in most participants in this study; however, other useful biomarkers for detecting LTBI, such as *Mtb* proteins Rv0222 and Rv3403c, *Mtb* thymidylate kinase antigen, and LAM, have been reported.^12^, ^13^, ^14^, ^15^ ESTA6 and Ag85A are expressed in growing bacilli, and Acr and MDP1 are upregulated in the stationary to dormant phases. The antibody responses against these antigens, which are associated with different growth states and correlated with bacterial burden,^16^, ^17^, ^18^ were strongest in patients with active TB, followed by those with recent LTBI (Figure 2). These results suggest that growing and dormant bacilli coexist in individuals with *Mtb* infection and that some, but not all, major antigens are useful antibody targets for determining the risk of TB progression. While TB granulomas differ in cellular composition and structure between patients with LTBI and those with active TB,^19^ different types of granulomatous lesions may be found in the same individual, either with active TB^20^ or with LTBI.^21^ In our previous study, immunohistochemistry revealed the colocalization of Ag85A, an antigen expressed by growing *Mtb*, and MDP1, an antigen expressed by stationary to dormant *Mtb*, inside tuberculous granuloma lesions in an asymptomatic individual with past TB, showing that *Mtb* in lesions can express both Ag85A and MDP1.^7^ It is conceivable that there are heterogeneous populations of dormant and multiplying tubercle bacilli within granulomatous lesions throughout the stages of TB infection, as observed in the analysis of past TB.

Identifying and treating (eg, isoniazid preventive therapy against growing bacilli, chemical prophylaxis) *Mtb*‐infected individuals with a risk of progression to active disease is crucial in achieving TB control.^22^, ^23^, ^24^ The five asymptomatic individuals with recent LTBI shown in Figure 3 had much higher antibody titers against all four antigens than those in the groups with remote LTBI and no *Mtb* infection. One individual among the five cases was later found to have developed active disease and was classified as preclinical TB. However, the present findings need validation in prospective, multicenter trials to determine whether such individuals are candidates for prophylactic therapy.

MDP1 has pleiotropic functions and is an essential protein for *Mtb*. It controls gene expression and suppresses mycobacterial multiplication under the hypoxic conditions of macrophages,^25^, ^26^ and also has ferritin‐like activity for controlling iron homeostasis.^27^ MDP1 stores iron inside bacteria and prevents the iron‐dependent generation of oxygen radicals.^27^ Iron is also essential for the survival and multiplication of *Mtb* inside macrophages. Our results and those of previous studies indicate that this protein is expressed more strongly in the latent stage than in the active disease state. This protein is likely involved in the decreased growth rate and long‐term persistence of *Mtb* in the latent state, thus accounting for IgG production in individuals with recent LTBI.

The Ag85 complex has mycolyl transferase enzymatic activity and mediates dynamic remodeling of mycolic acid‐containing glycolipids in the cell wall via its broad substrate specificity.^28^, ^29^, ^30^ These immune responses to glycerol monomycolate occur specifically in people with LTBI, but not in patients with active TB.^31^ Thus, Ag85‐dependent exchange of trehalose 6‐monomycolate to glycerol monomycolate is likely to occur in LTBI. It is not surprising that Ag85 may be useful for detecting asymptomatic *Mtb* infection with a risk of TB progression. It may be possible to identify individuals at high‐risk of preclinical or active TB by assessing IgA and/or IgG responses to the other *Mtb* protein antigens.^32^

Although ESAT6, CFP10, and Acr are *Mtb*‐specific antigens, antibodies against Ag85A and MDP1 are produced in the sera of patients with pulmonary MAC disease; these antigens exist in other acid‐fast bacilli (data not shown). A patient who was excluded on the basis of a MAC test result in this study was later diagnosed with MAC disease. To accurately detect recent LTBI, it is necessary to exclude NTM infection using biomarkers with antigenicity that is specific to other acid‐fast bacteria, such as MAC‐specific glycopeptide lipid.^33^

Accurate detection of recent LTBI/preclinical TB status and progression from LTBI to active TB disease can be achieved by assessing both humoral and cellular immune responses with combinations of characteristic antigens, including growth‐related ESAT6, CFP10, Ag85A, dormancy‐related MDP1, and Acr. Accurate detection will facilitate more effective preventive therapy and reduction in disease incidence, paving the way for eradication of TB. This study was performed at a single facility. Although cases with recent LTBI were accurately identified and followed regularly for 6 months, the sample size was small. Further larger multicenter trials are needed.

## CONFLICTS OF INTERESTS

The authors declare that they have no conflict of interests.
